# Long-Term Return to Work After Acquired Brain Injury in Young Danish Adults: A Nation-Wide Registry-Based Cohort Study

**DOI:** 10.3389/fneur.2018.01180

**Published:** 2019-01-14

**Authors:** Maiken Tibæk, Lars Peter Kammersgaard, Søren P. Johnsen, Christian Dehlendorff, Hysse B. Forchhammer

**Affiliations:** ^1^National Study of Young Survivors of Brain Injury, Department of Neurology, Rigshospitalet, University of Copenhagen, Copenhagen, Denmark; ^2^Research Unit on Brain Injury Rehabilitation Copenhagen, Department of Neurorehabilitation, TBI Unit, Rigshospitalet, University of Copenhagen, Copenhagen, Denmark; ^3^Department of Clinical Epidemiology, Aarhus University Hospital, Aarhus, Denmark; ^4^Statistics and Pharmacoepidemiology, Danish Cancer Society Research Center, Copenhagen, Denmark

**Keywords:** brain injury, return to work, employment, prognosis, TBI, young adults

## Abstract

**Objective:** (1) To determine patterns of return to work (RTW) after traumatic brain injury and other causes of acquired brain injury (ABI) among young adults aged 19–30 years and (2) to compare the stability of long-term labor-market attachment (LMA) to the background population.

**Method:** Nationwide registry-based inception cohort study of 10 years weekly data of employment status. Patients (*n* = 8,496) aged 19–30 years with first-ever diagnosis of TBI, stroke, subarachnoid hemorrhage, encephalopathy, brain tumor, or CNS infections during 1999–2015. For comparison, a general population cohort (*n* = 206,025) individually matched on age, sex, and municipality was identified. The main outcome was RTW, which was defined as time to LMA, i.e., a week without public assistance benefits except education grants/leave. Stable labor-market attachment (sLMA) was defined as LMA for at least 75% over 52 weeks. The cumulative incidence proportions of RTW and stable RTW in the ABI cohort were estimated with the Aalen-Johansen estimator with death as a competing event.

**Results:** Twelve weeks after diagnosis 46.9% of ABI cohort had returned to stable RTW, which increased to 57.4% 1 year after, and 69.7% 10 years after. However, compared to controls fewer had sLMA 1 year (OR: 0.25 [95% CI 0.24–0.27]) and 10 years after diagnosis (OR: 0.35 [95% CI: 0.33–0.38]). Despite significant variations, sLMA was lower compared to the control cohort for all subtypes of ABI and no significant improvements were seen after 2–5 years.

**Conclusion:** Despite relatively fast RTW only a minor proportion of young patients with ABI achieves sLMA.

## Introduction

Acquired brain injury (ABI) is by definition an injury to the brain that is not hereditary, congenital, or degenerative, but acquired after birth. ABI constitutes a variety of injuries such as traumatic brain injury (TBI) and non-traumatic causes (non-TBI), where the latter includes stroke, subarachnoid hemorrhage (SAH), CNS infection, encephalopathy, and brain tumor (1). ABI is among the most disabling conditions (2, 3) with potentially profound implications for the individual, family, and society, since it often leads to short- and long-term physical, communicative, cognitive, and emotional dysfunction. Consequently, the capability to return to work (RTW) or resume education (4–6) may be reduced, since it is highly dependent of physical, cognitive, communicative, and emotional functioning.

Young adults often have better functional outcomes after TBI or stroke compared to older adults (7, 8). Nevertheless, younger age is reported as a negative predictor of long-term outcome such as RTW (5, 9). Young adults are often in the process of completing their educational achievements or are in the early stages of their careers. Since ABI may influence or even hamper the ability to complete education or maintain stable labor-market attachment (LMA), young adults may be a particularly vulnerable group (5, 10).

Return to work ability after ABI is largely unknown, but some reports are available for stroke and TBI. For TBI patients irrespectively of age, 30.5% has been reported to RTW 1 year after injury (11), whereas 11.0–59.5% (12) of younger stroke patients RTW. Corresponding figures for SAH were 35.2–71.5% (13, 14), whereas data are limited for patients with brain tumor (15) and unknown for encephalopathy. For TBI, it has been shown that only a minor proportion achieves stable LMA (sLMA) despite the fact that many at some point return to work (5, 16). Furthermore, the cause of injury i.e., TBI or non-TBI is reported not to influence the RTW (11). However, none of these studies are nationwide or compare to an age-matched background population.

The primary objective of this study was to determine the likelihood of RTW after ABI among young adults in Denmark aged 19–30 years for up to 10 years following injury and to compare RTW in different subtypes of ABI. Furthermore, to compare sLMA among ABI patients to the general population.

## Materials and Methods

Two cohorts were identified using nationwide population-based Danish registries. (1) All Danish patients between 19 and 30 years diagnosed with a first-ever stroke, traumatic brain Injury, brain tumor, encephalopathy, CNS infection, or SAH during the period 1999–2013. (2) A comparison cohort extracted from the general Danish population and individually matched to the ABI patients on age, sex, and municipality.

### Setting

In 2015 Denmark had a population of 883,909 young adults aged 19–30 years. Employment rate in this age-group increased with age from 49.3 to 69.3% (17). The government provides financial support in case of sickness or unemployment. Sickness benefits are available for a limited period or until the individual returns to work. A person must be actively job seeking to receive unemployment benefits and the benefits are reduced after prolonged sickness or unemployment. All Danish students regardless of social status are entitled to a state educational grant from the age of 18 years if they attend a youth education program (high school level) or higher education. The healthcare-system in Denmark is primarily publicly funded and all patients with the diagnoses included are exclusively treated by publicly hospitals during the acute phase.

### Data Sources

The Danish Civil Registration System holds information on immigration, municipality at index, death, sex, and date of birth for all Danish citizens (18). The unique personal identification number assigned to all Danish citizens was used for accurate individual-level linkage between registries. The Danish National Registry of Patients (DNP) covers all contacts to Danish public hospitals. DNP holds information on all admissions since 1977 and all outpatient contacts since 1994 (19). Diagnoses have been classified according to the International Classification of Disease revision 10 (ICD-10) since 1994.

Employment status was obtained from the DREAM-registry administered by the Danish National Labor-market Authority (20), which provided weekly information of self-support as well as any public transfer payments including state education grants given to all persons aged 18–65 years with a Danish civil registration number. We used the socio-economic background of the participants' mothers. The mothers were identified in the fertility database (21) and subsequently highest attained educational level (basic school, high school, short education, higher education, or unknown) from the Danish Educational attainment Registry (22) and age-adjusted quintiles of disposable income from registries administered by Statistics Denmark were obtained. Finally, cohort members were classified as immigrants, descendants or native Danes by using information from Statistics Denmark (23).

### Population

The ABI patients were identified in DNP and were required to have a first-ever diagnosis of brain tumor, CNS infection, encephalopathy, stroke, SAH, or TBI between 1999 and 2013 (Table 1). Patients only seen at out-patient clinics were not included. In addition, we excluded cases from the period 1994–1998 to ensure that cases during 1999–2013 were first-ever cases.

**Table 1:** ICD-10 codes.

**Diagnosis**	**ICD-10 code**
Stroke	I60-1, I63-4, DI679, DI67-DI68 (-I674), DG46
Traumatic brain injury	S020, S021, S027-S029, S061-S071, S097, T020, T040, T060
Encephalopathy (anoxic/metabolic diseases)	B220, E159, E512, G410, G929, G931, G938, G978, I460, O292, O743, O754, O892, T58, T719, T751, I674, G 372
CNS infections	A321, A390, A398, B003, B004, G040, G042, G048, G05, G060, G07-G09
Brain tumor	C70-C71, D32, D330, D332, D337, D339,

The general population comparison cohort was identified using the Danish Civil Registration System by risk set sampling, i.e., controls had to be alive and at risk of first-ever ABI at the date of diagnosis (index) of the corresponding ABI patient. For each ABI patient 25 individuals matched by age (month and year), sex, and municipality at index were selected. The matching factors were chosen a priori to address confounding by age, sex, and regional differences in socio-economic position and unemployment rates. Both patients and controls were required to be residing in Denmark 1 year prior to index. Patients and controls were followed from 12 weeks after index until December 31, 2015, death or emigration, whichever came first.

### Study Outcomes

For each week, individuals were defined as self-supportive if no public benefits, except for state education grant, were received or while being on leave. If an individual was self-supportive for at least 75% of 52 consecutive weeks they were considered to have a stable labor-marked attachment (sLMA) (24). This cut-off was chosen to require the individuals to be primarily self-supportive.

For the ABI cohort, time to RTW was defined as the first week of self-supportiveness after baseline (12 weeks after the diagnosis). Time to stable return to work (sRTW) was defined as the time from baseline to the first week followed by of a 52-week period with self-supportiveness in at least 75% of the period.

### Statistical Analysis

SLMA for the ABI-cohort was compared with the general population cohort at 1, 2, 5, and 10 years after baseline by conditional logistic regression accounting for the matching and corresponding odds ratios (OR) and 95% confidence intervals (CI) estimated. Two types of adjustment were done (1) according to the matching (simple adjustment) and (2) with further adjustment for sLMA 1-year prior to index, immigration status, mothers' income and mothers' highest achieved educational level (full adjustment). Individuals that had died or emigrated at a given time-point were excluded from that analysis. In addition, we only considered complete cases, however, treating missing values as a separate category, did not change the estimates materially (data not shown). Furthermore, we did two sensitivity analyses: (1) excluding cases identified only through emergency room contacts and (2) using alternative cut-offs for defining stable LMA, i.e., >50 and >90%, respectively.

The cumulative incidence proportions of RTW and sRTW in the ABI cohort were estimated with the Aalen-Johansen estimator with death as competing event (25). Significance level was set to 5% and all analysis were performed in “R version 3.2.3” (26). The study was approved by the Danish Data Protection Agency. Ethical approval or individual consent was not required for this type of study by Danish legislation.

## Results

### Descriptive Data

Of the 8,496 patients who met the inclusion criteria, 4,645 (54.7%) had TBI, 1,333 (15.7%) had stroke, 575 (6.8%), had SAH, 829 (9.8%) had encephalopathy, 634 (7.5%) had CNS infection and 480 (5.6%) had brain tumor. Some differences were seen between the ABI cohort and controls (Table 2). Both the mother's income and educational level were lower in the ABI cohort. The proportion of immigrants and descendants were significantly higher in the ABI cohort compared to the general population cohort. During the study 393 (4.6%) died and 398 (4.7%) emigrated within the ABI cohort, whereas 1,151 (0.6%) died and 12,258 (5.9%) emigrated within the comparison cohort.

**Table 2:** Descriptive characteristics.

**Variables**	**ABI cohort**		**Control cohort**	
	***N***	**%**	***N***	**%**	***p*****-value**
Total	8,496		206,025		
Female sex	3,189	37.5	77,040	37.4	
Mean age at diagnose (sd)	24.40	3.54	24.40	3.55	
**Overall**					
Brain tumor	480	5.6	11,642	5.7	
CNS infection	634	7.5	15,317	7.5	
Encephalopathy	829	9.8	20,075	9.8	
SAH	575	6.8	13,915	6.8	
Stroke	1,333	15.7	32,285	15.7	
TBI	4,645	54.7	112,794	54.7	
Self-supportive 1 year before injury	5,677	66.8	168,326	81.7	<0.001
Mother's disposable income					<0.001
0–20%	1,810	21.3	35,556	17.3	
20–40%	1,552	18.3	34,909	16.9	
40–60%	1,409	16.6	36,803	17.9	
60–80%	1,518	17.9	39,879	19.4	
>80%	1,481	17.4	39,160	19.0	
Missing	726	8.5	19,721	9.6	
Highest educational level of mother					<0.001
Basic school	2,915	34.3	61,455	29.8	
High school	212	2.5	5,236	2.6	
Low education (incl vocational education)	2,841	33.4	71,915	34.9	
Higher education	1,576	18.5	43,485	21.1	
Missing	952	11.2	23,910	11.2	
Immigration status					<0.001
Danish	7,614	89.6	182,779	88.7	
Descendants	667	7.9	18,648	9.1	
Immigrants	215	2.5	4,601	2.2	

### Return to Work

**Figure 1:**
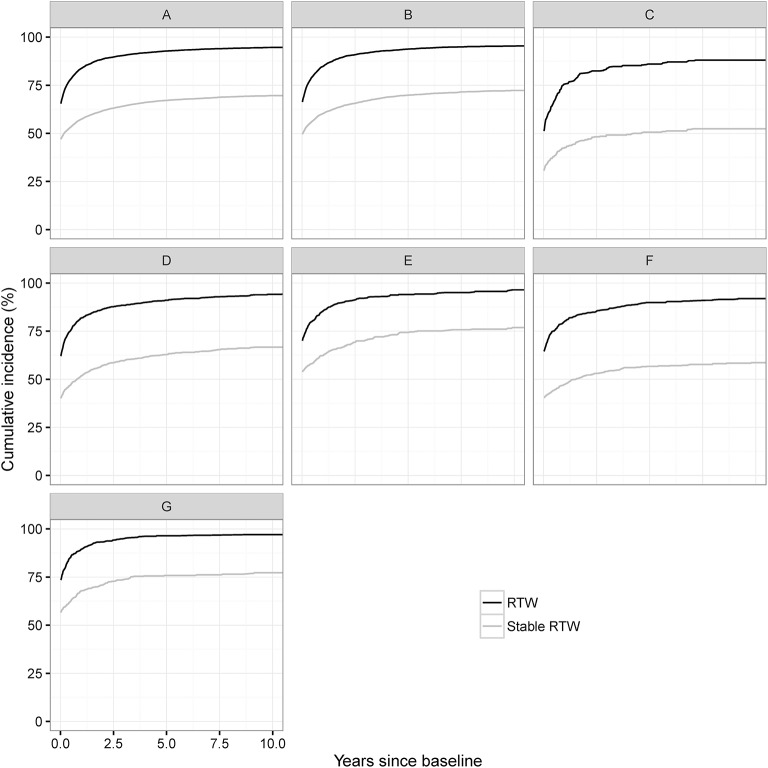
Cumulative incidence of RTW and sRTW according to type of brain injury: **(A)** ABI cohort, **(B)** TBI, **(C)** Brain Tumor, **(D)** Stroke, **(E)** SAH, **(F)** Encephalopathy, and **(G)** CNS infection.

The cumulative incidences of RTW and stable RTW are shown in Figure 1 and in Supplementary Table 1. Across diagnoses 50–75% had returned to work at baseline, the corresponding numbers for sRTW were 30–60%. Overall, the cumulative incidence of sRTW mainly increased during the first 5 years, while RTW and sRTW reached levels between 88–95 and 55–80% after 10 years for RTW and sRTW, respectively. Brain tumors had the lowest cumulative incidence of stable RTW followed by encephalopathy, and stroke, whereas CNS infection had the highest proportion of RTW and stable RTW followed by SAH and TBI. Persons with sLMA 1-year prior to index had a higher likelihood of stable RTW (data not shown). Excluding emergency room cases gave slightly lower RTW and sRTW however with similar patterns as in the main analysis (data not shown).

### Labor-Market Attachment

**Figure 2:**
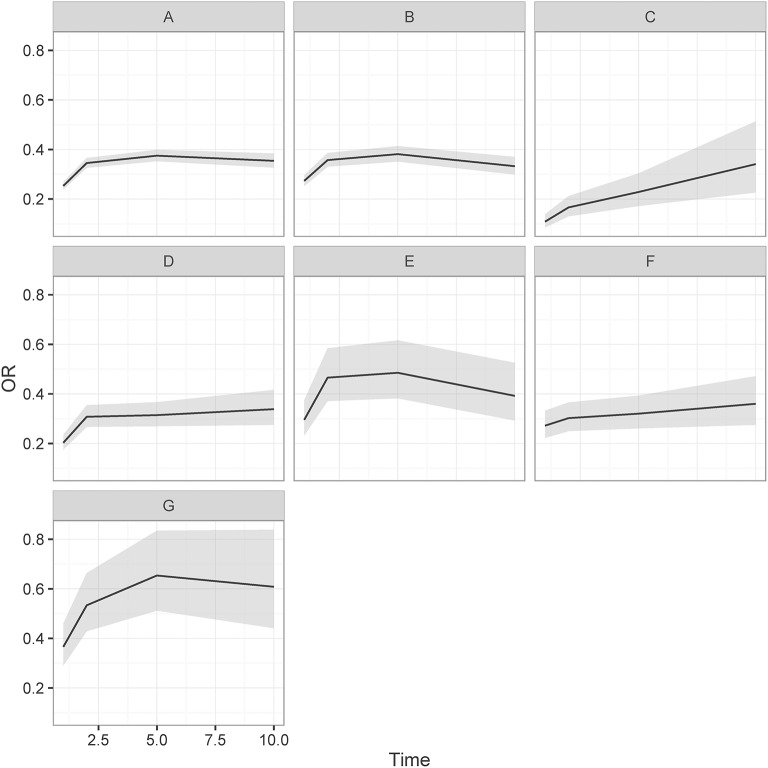
Adjusted odds ratio and CI (gray area) for stable labor market attachment for ABI cohort compared to matched controls: **(A)** ABI cohort **(B)** TBI, **(C)** Brain Tumor, **(D)** Stroke, **(E)** SAH, **(F)** Encephalopathy, and **(G)** CNS infection.

Simple and fully adjusted odds ratios for sLMA for the ABI cohort compared to the matched controls are given in Table 3. The fully adjusted odds ratio for 1 year (aOR_1YR_), 2 years (aOR_2YR_), 5 years (aOR_5yr_), and 10 years (aOR_10yr_) are shown in Figure 2. The proportion of the control cohort with sLMA was 77.4–85.7% throughout the follow-up period (Table 3). In the ABI cohort 52.9% had sLMA 1 year after baseline, which was significantly lower compared to the control cohort (aOR_1yr_: 0.25 [95% CI 0.24–0.27]). Ten years after baseline the proportion of sLMA in the ABI cohort had increased slightly to 53.2% but remained significantly lower compared to the controls (aOR_10YR_ 0.35 [95% CI: 0.33–0.38]). Despite significant variations in the proportion of patients with sLMA between diagnoses, sLMA remained significantly lower compared to the control cohort for all diagnoses. Most of the diagnoses (i.e., TBI, SAH, stroke, encephalopathy, and CNS infections) showed no improvements in LMA from 2 to 5 years after baseline. However, after 2 years CNS infections had the highest OR for sLMA compared to control cohort (aOR_2YR_: 0.53 [95% CI: 0.43–0.66]), followed by SAH (aOR_2YR_: 0.47 [95% CI: 0.37–0.58]), TBI (aOR_2YR_: 0.36[95% CI: 0.33–0.39]), stroke (aOR_2YR_: 0.31 [95% CI: 0.27–0.35]), encephalopathy (aOR_2YR_: 0.30 [95% CI: 0.25–0.37]), and brain tumor (aOR_2YR_: 0.17 [95% CI: 0.13–0.21]). In the sensitivity analyses of defining sLMA using 50 and 90% cut-offs, respectively, no significant changes were seen (data not shown). Similarly, excluding emergency room contacts yielded similar conclusions (data not shown). Only SAH had lower ORs after excluding emergency room contacts (aOR_5YR_: 0.32 [95% CI: 0.23–0.45]) and when defining sLMA using 50% as cut-off (aOR_5YR_: 0.39 [95% CI: 0.30–0.50]), respectively.

**Table 3:** Stable labor market attachment 1–10 years after ABI diagnosis.

	**1 year before index**			**1 year after baseline**							**2 years after baseline**							
**Diagnosis**	**ABI n (%)**	**Controls *n* (%)**	**ABI *n* (%)**	**Controls *n* (%)**	**Crude OR**	**Crude 95% CI**		**Adj OR**	**Adj. 95% CI**		**ABI n (%)**	**Controls *n* (%)**	**Crude OR**	**Crude 95% CI**		**Adj OR**	**Adjusted 95% CI**	
ABI	5,848 (66.3)	181,128 (81.7)	4,433 (52.93)	175,801 (80.61)	0.26	0.25	0.27	0.25	0.24	0.27	4,534 (55.95)	169,167 (79.8)	0.31	0.29	0.32	0.35	0.33	0.37
TBI	3,292 (68.8)	99,675 (82.3)	2,543 (55.35)	96,680 (81.9)	0.28	0.26	0.29	0.27	0.25	0.30	2,590 (58.23)	93,011 (80.4)	0.33	0.31	0.35	0.36	0.33	0.39
Brain tumor	327 (65.7)	9,892 (80.6)	173 (37.7)	9,621 (79.5)	0.14	0.12	0.18	0.11	0.09	0.14	182 (43.0)	9,251 (79.1)	0.19	0.16	0.24	0.17	0.13	0.21
SAH	417 (70.1)	12,065 (81.1)	337 (59.0)	11,757 (80.4)	0.33	0.28	0.39	0.30	0.23	0.37	345 (61.7)	11,267 (79.2)	0.41	0.34	0.49	0.47	0.37	0.58
Stroke	898 (64.2)	27,875 (80.5)	627 (47.5)	27,122 (79.7)	0.22	0.19	0.24	0.20	0.18	0.24	664 (52.1)	26,097 (78.8)	0.28	0.25	0.32	0.31	0.27	0.35
Encephalopathy	444 (49.5)	18,390 (80.8)	364 (44.9)	17,846 (79.8)	0.19	0.16	0.22	0.27	0.22	0.33	354 (45.2)	17,232 (79.2)	0.2	0.17	0.23	0.30	0.25	0.37
CNS infection	470 (72.2)	13,231 (82.3)	389 (62.6)	12,775 (80.7)	0.38	0.32	0.45	0.37	0.29	0.46	399 (65.7)	12,309 (80.0)	0.47	0.40	0.57	0.53	0.43	0.66
	**5 years after baseline**								**10 years after baseline**									
	**ABI n (%)**	**Controls n (%)**	**Crude OR**	**Crude 95% CI**		**Adj OR**	**Adjusted 95% CI**		**ABI n (%)**	**Controls n (%)**	**Crude OR**	**Crude 95% CI**		**Adj OR**	**Adjusted 95% CI**			
ABI	3,457 (54.95)	130,952 (78.8)	0.32	0.31	0.34	0.37	0.35	0.40	1,872 (53.16)	74,033 (78.0)	0.32	0.29	0.34	0.35	0.33	0.38		
TBI	1,980 (56.1)	72,410 (79.1)	0.33	0.31	0.36	0.38	0.35	0.41	1,028 (51.78)	40,980 (78.0)	0.3	0.27	0.33	0.33	0.30	0.37		
Brain tumor	125 (46.1)	7,026 (78.8)	0.22	0.17	0.28	0.23	0.17	0.30	66 (49.1)	4,090 (78.9)	0.24	0.17	0.34	0.34	0.23	0.51		
SAH	283 (62.6)	9,090 (79.3)	0.43	0.35	0.52	0.49	0.38	0.62	168 (58.5)	5,645 (78.00)	0.38	0.30	0.48	0.39	0.29	0.53		
Stroke	496 (50.4)	20,014 (78.0)	0.28	0.25	0.32	0.31	0.27	0.37	270 (52.6)	10,527 (77.4)	0.32	0.26	0.38	0.34	0.28	0.42		
Encephalopathy	269 (44.5)	13,443 (78.5)	0.21	0.18	0.25	0.32	0.26	0.39	171 (48.6)	7,635 (85.8)	0.26	0.21	0.32	0.36	0.28	0.47		
CNS infection	304 (68.0)	8,969 (78.2)	0.59	0.48	0.73	0.65	0.51	0.83	169 (67.9)	5,156 (77.9)	0.6	0.45	0.79	0.61	0.44	0.84		

## Discussion

This study shows that ABI has a major impact on RTW and especially on sLMA even 10 years after ABI. Overall, the proportion of ABI patients with sLMA increases until 2 years post-injury with no further significant improvement thereafter. One major exception was, however, for brain tumor patients, who showed increasing sLMA even in the subsequent years. Even though up to 95% of the ABI cohort return to work or education, and up to 77% achieve 1 year of sLMA at some point during the 10 years follow up, only half of the ABI cohort have sLMA after 10 years. This study is, to our knowledge, the first to present nationwide data on RTW and sRTW, as well as sLMA for young adults covering the full spectrum of ABI. Most studies only report data for TBI patients and to a lesser extent stroke. Furthermore, most published RTW studies use a cross-sectional design, which is a design that may not fully capture absence of sLMA. Additionally, direct comparison should be performed with caution, because of substantial differences in setting, case ascertainment, study design, data collection, and outcome definitions across studies (27, 28).

With regard to TBI, large variations (between 30 and 65%) in the proportion that RTW after brain injury (11) have been reported. In settings, comparable to Denmark, studies from Australia and Norway have reported markedly lower levels of RTW compared to our results with RTWs between 45 and 55%, respectively (16, 29, 30). However, patients in the Australian and Norwegian studies were recruited from intensive care units or rehabilitation facilities and are therefore probably more severe cases, whereas we included all cases (including mild cases) in Denmark. The diverging results are thus most likely explained by differences in severity and that this study only considers young adults.

For stroke, specific data regarding the young adults are even more sparse than for TBI. Much in line with our results, a comprehensive review showed that the majority of the stroke patients return to work within the first year, and often within the first 3–6 months (28). However, patients included the entire working age spectrum and large variations with respect to age were reported (19–73%). A Swedish study reported that 74.4% of 18–34 year-old stroke patients had returned to work (both full-time and part-time considered) and the majority within the first 12 months after the stroke (31). This result is higher than that found in our study, and is probably caused by different definitions of RTW.

For patients with glioblastoma the proportion that RTW after 1 year has been reported to be 27% (15), which is lower compared to RTW for brain tumor patients in our study. This difference is likely caused by the higher mortality and higher levels of impairments following glioblastoma compared to the less aggressive tumors that predominates in our young cohort. Also, this may suggest that brain tumors in this age-group often cause a gradually progressing brain injury than the remaining diagnoses considered in this study. On the other hand our results are in line with a British study, which reported a RTW proportion of 52% in a cohort of brain tumor patients recruited from a rehabilitation facility (32).

To our knowledge no reports have presented results of the cumulative incidence of sRTW after ABI. Some studies have explored sLMA after TBI (5, 16, 33) and found that a significant proportion of those who RTW have unstable LMA. This finding is in line with our results, where we found a similar tendency for all diagnoses considered. In a review by van Velzen et al. the authors concluded that the level of RTW was similar for traumatic and non-traumatic ABI after the initial first year (11). However, in this study we found important differences in RTW between the diagnoses that constitute non-traumatic ABI, namely that brain tumor, encephalopathy and CNS infections were significantly different from TBI in terms of RTW. All three subgroups are, however, small compared to the stroke group, and a pooled analysis of non-traumatic ABI group would tend to be dominated by stroke patients.

Although most developed countries provide sickness benefit, comparison across countries should be done with caution due to country-specific requirements and level of compensation. Despite the favorable Danish benefits, individuals are nevertheless focused on returning to work since sickness benefits are only available for limited time. It is therefore notable that despite the relatively favorable conditions for long-term sickness compared to other countries, RTW is nevertheless relatively high in Denmark.

### Study Limitations

The main strengths of our study are the nation-wide design, the use of a comparison cohort and the long-term data of public benefits. Furthermore, the unique Danish personal identification number enabled us to link accurately between medical data and employment data. Nonetheless, registry studies are at risk of misclassification and in the current study we relied on the accuracy of discharge-diagnoses and DREAM-data. The discharge data are generally of high quality, however only the validity of stroke and specific CNS infections are known (19). For both diagnoses some misclassification may have occurred in up to 15–20% of the diagnoses and for emergency room contacts even higher (34). However, excluding emergency room contact gave similar patterns although slightly lower LMA. Employment data were retrieved from DREAM, which can elucidate the dynamics of RTW because of the weekly individual registrations (24). Data in the registry have a high positive predictive value (20, 35), however, only sick leaves longer than 2 weeks are registered if the employer claims a reimbursement fee (20). Also, misclassification may have occurred for persons living on own assets or support from family, but this is rare in Denmark. Preexisting comorbidity may also decrease RTW, but to overcome this bias, we adjusted for sLMA prior to index to account for pre-injury differences in LMA between the cohorts. Another limitation to this study is that we did not have information on the severity of ABI. Largely, because there is no uniform measure of injury severity across different types of ABI and such information was not systematically registered for all the included diagnoses in Denmark. In addition, structural factors such as economic recession and structural changes in the Danish health care sector might also have influenced the results. But these factors were not likely to influence the results because the OR for sLMA stratified by year of diagnosis showed no significant differences (data not shown). Furthermore, we might have overestimated the 5- and 10-year estimates for LMA among brain tumor patients, since in contrast to the rest of the ABI cohort, the mortality after brain tumor increases even 5–10 years after index leaving primarily individuals with less severe impairments. A significant proportion of the diagnoses included in this study is associated with low social status (36, 37), which also influences the ability to RTW and subsequent sLMA. Thus, one may overestimate the impact of brain injury if this selection bias is ignored. This problem was addressed by matching and adjusting for social status and consequently we believe this bias is small. Furthermore, socio-economic background of the participants' mothers was used since the socio-economic position of a young adult may be better reflected by the mother's data than by the person's own data in this age-group. However, we cannot rule out some degree of residual confounding, but it is unlikely that it explains the difference in sLMA in ABI patients and their population controls. Finally, the cut-off for sLMA of 75% favors a conservative estimate of the impact of ABI on the ability to assume work.

## Conclusion

This study demonstrates that a large proportion of young ABI patients return to work within 1 year following brain injury, whereas many fail to achieve long-term sLMA. Young brain tumor patients, however, appear to steadily increase long-term LMA. These findings underline that vocational rehabilitation following acquired brain injury is a long-lasting rather than just a temporary effort to maintain labor market attachment.

## Author Contributions

MT, LK, SJ, CD, and HF designed and planned the study. MT and CD assembled the data, performed the statistical analyses, and drafted the manuscript. All authors discussed the results and contributed to the final manuscript.

### Conflict of Interest Statement

The authors declare that the research was conducted in the absence of any commercial or financial relationships that could be construed as a potential conflict of interest.

## Abbreviations

ABI: Acquired Brain Injury
CI: Confidence Interval
CNS: Central Nervous System
LMA: Labor-market attachment
DNP: Danish National Registry of Patients
OR: Odds Ratio
RTW: Return To Work
SAH: Subarachnoid Hemorrhage
sLMA: Stable Labor-market attachment
sRTW: Stable Return To Work
TBI: Traumatic Brain Injury.
