# Supplementary material for: Long-Term Return to Work After Acquired Brain Injury in Young Danish Adults: A Nation-Wide Registry-Based Cohort Study
Source: Front Neurol. 2019 Jan 14;9:1180. doi: 10.3389/fneur.2018.01180 (PMC6340062; doi:10.3389/fneur.2018.01180)
Supplement: Supplementary file 1 [file Table_1.pdf]

Supplementary material to

Long-term return to work after acquired brain injury in young Danish adults: a nation-wide registry-based cohort study

Maiken Tibæk, Lars P. Kammersgaard, Søren P. Johnsen, Christian Dehlendorff and Hysse B. Forchhammer

Content:

Table S1: Cumulative incidence of return to work and stable return to work

Supplementary Table 1:

		Return to work			Stable return to work		
	years after baseline	cumulative incidence of RTW	95% CI		cumulative incidence of stable RTW	95% CI	
ABI	Baseline	65.44	64.4	66.48	46.94	45.88	48.01
ABI	1	83.86	83.05	84.66	57.43	56.38	58.49
ABI	2	88.6	87.89	89.29	61.81	60.77	62.84
ABI	5	92.77	92.18	93.34	67.13	66.11	68.15
ABI	10	94.65	94.09	95.17	69.65	68.61	70.68
TBI	Baseline	66.28	64.88	67.67	49.53	48.1	50.97
TBI	1	84.87	83.8	85.91	60.33	58.92	61.74
TBI	2	89.88	88.96	90.75	64.57	63.19	65.95
TBI	5	93.76	93	94.46	69.87	68.51	71.21
TBI	10	95.37	94.67	96.01	72.29	70.92	73.64
Brain tumor	Baseline	51.22	46.7	55.93	30.69	26.77	35.03
Brain tumor	1	75.5	71.44	79.37	42.64	38.35	47.21
Brain tumor	2	81.29	77.55	84.75	46.66	42.3	51.24
Brain tumor	5	86.01	82.59	89.06	50.61	46.15	55.24
Brain tumor	10	88.07	84.77	90.95	52.36	47.81	57.07
Stroke	Baseline	62.06	59.35	64.78	40.14	37.56	42.82
Stroke	1	81.93	79.72	84.03	51.82	49.16	54.53
Stroke	2	86.44	84.45	88.29	57.24	54.59	59.91
Stroke	5	91.06	89.33	92.61	62.77	60.13	65.42
Stroke	10	94.1	92.48	95.47	66.72	63.98	69.43
SAH	Baseline	70.04	66.19	73.8	53.83	49.81	57.96
SAH	1	85.56	82.5	88.34	62.37	58.42	66.33
SAH	2	90.11	87.44	92.42	66.94	63.08	70.77
SAH	5	94.03	91.82	95.81	74.34	70.59	77.94
SAH	10	96.46	94.32	97.96	76.87	73.06	80.5
Encephalopathy	Baseline	64.52	61.09	67.94	40.58	37.32	44.01
Encephalopathy	1	80.19	77.26	82.96	47.4	44.06	50.86
Encephalopathy	2	84.21	81.5	86.72	51.96	48.6	55.41
Encephalopathy	5	89.87	87.51	91.95	56.72	53.32	60.17
Encephalopathy	10	91.93	89.66	93.87	58.65	55.17	62.17
CNS infection	Baseline	73.5	69.96	76.92	56.62	52.81	60.51
CNS infection	1	89.5	86.92	91.77	67.92	64.25	71.54
CNS infection	2	93.11	90.92	94.94	70.88	67.28	74.39
CNS infection	5	96.46	94.69	97.77	75.84	72.36	79.18
CNS infection	10	97.1	95.32	98.33	77.3	73.72	80.7
